# The *Listeria monocytogenes* strain 10403S BioCyc database

**DOI:** 10.1093/database/bav027

**Published:** 2015-03-28

**Authors:** Renato H. Orsi, Teresa M. Bergholz, Martin Wiedmann, Kathryn J. Boor

**Affiliations:** Department of Food Science, Cornell University, Ithaca, NY 14853, USA

## Abstract

*Listeria monocytogenes* is a food-borne pathogen of humans and other animals. The striking ability to survive several stresses usually used for food preservation makes *L. monocytogenes* one of the biggest concerns to the food industry, while the high mortality of listeriosis in specific groups of humans makes it a great concern for public health. Previous studies have shown that a regulatory network involving alternative sigma (σ) factors and transcription factors is pivotal to stress survival. However, few studies have evaluated at the metabolic networks controlled by these regulatory mechanisms. The *L. monocytogenes* BioCyc database uses the strain 10403S as a model. Computer-generated initial annotation for all genes also allowed for identification, annotation and display of predicted reactions and pathways carried out by a single cell. Further ongoing manual curation based on published data as well as database mining for selected genes allowed the more refined annotation of functions, which, in turn, allowed for annotation of new pathways and fine-tuning of previously defined pathways to more *L. monocytogenes*-specific pathways. Using RNA-Seq data, several transcription start sites and promoter regions were mapped to the 10403S genome and annotated within the database. Additionally, the identification of promoter regions and a comprehensive review of available literature allowed the annotation of several regulatory interactions involving σ factors and transcription factors. The *L. monocytogenes* 10403S BioCyc database is a new resource for researchers studying *Listeria* and related organisms. It allows users to (i) have a comprehensive view of all reactions and pathways predicted to take place within the cell in the cellular overview, as well as to (ii) upload their own data, such as differential expression data, to visualize the data in the scope of predicted pathways and regulatory networks and to carry on enrichment analyses using several different annotations available within the database.

**Database URL**: http://biocyc.org/organism-summary?object=10403S_RAST

## Introduction

*Listeria monocytogenes* is a food-borne bacterial pathogen and the etiological agent of a disease known as listeriosis, which affects humans and other animals. Although listeriosis is somewhat rare, with an estimated number of approximately 1590 cases per year in USA (1), it has been estimated that 15.9 % of infected people die from this disease (1) making *L. monocytogenes* the third most common cause of food-borne disease related deaths, representing 19 % of all food-borne disease related deaths in USA (1). Therefore, considerable efforts have focused on minimizing the risks of infection, both through improved strategies to prevent contamination of Ready-To-Eat foods with *L. monocytogenes* and through improved human disease surveillance. However, *L. monocytogenes* continues to be a threat to human health and to the food industry with an estimated economic cost of US$1.2 billion to US$2.4 billion due to listeriosis yearly in the US (2). The major difficulty imposed by *L. monocytogenes* to the food industry is the striking ability this pathogen has to survive and grow under environmental conditions usually used to preserve foods, such as low temperature, high osmolarity and low pH (3–5). Importantly, several regulatory pathways and stress response systems used by *L. monocytogenes* to overcome harsh conditions are also used during host infection and it has been shown that exposure to nonlethal stress conditions may actually enhance the virulence of certain strains (6–8).

Among the many factors that contribute to both stress survival and virulence is the general stress-response alternative sigma (σ) factor σ^B^. σ^B^ is the most studied among the four alternative σ factors (i.e. σ^B^, σ^C^, σ^H^ and σ^L^) found in *L. monocytogenes* and also has the largest regulon. The other three alternative σ factors, σ ^C^, which is specific to lineage II strains, σ^H^ and σ^L^ are thought to be involved in more specific stress responses and to regulate a smaller number of genes (9, 10). Alternative σ factors have been associated with response to several stresses including nutrient scarcity, osmotic stress, acid stress, cold stress and oxidative stress and together regulate more than 150 genes. Although several studies have assessed alternative σ factor regulons, little has been done to interpret this data in the light of metabolic functions, reactions and pathways carried out by their regulatees. Moreover, although *L. monocytogenes* is an important pathogen and has been used as a model organism for immune response studies to intracellular pathogens (11), few resources in terms of the functional genomics of this organism are currently available to researchers. Among current databases dedicated to *L. monocytogenes* is the GenoList (12), which provides access to several genomic features for strain EGD-e including access to gene and protein sequences, sequence analyses tools such as BLAST searches, pattern searches, comparative analysis for presence/absence of genes and proteins, and also includes a synteny viewer. The GECO database (13) also supports *L. monocytogenes* genomes (i.e. strain EGD-e and F2365). This database was designed for comparative genomics analyses and allows user to (i) detect genes that were horizontally transferred and pseudogenes and to (ii) assess the gene content of genomes in comparison to other genomes (13). The LEGER database (14) is another *Listeria*-specific database available, which provides information on gene functions, protein expression levels from published experiments, subcellular localization of proteins and the biological significance of genes and proteins based on the Kyoto Encyclopedia of Genes and Genomes (KEGG), InterPro and gene ontology (15). The Kyoto Encyclopedia of Genes and Genomes (16) also supports a *L. monocytogenes* web page with access to pathways and modules computationally generated (17). Besides these more comprehensive databases, task-specific *L. monocytogenes* databases have been designed and made available for Multilocus Sequence Typing (MLST) studies (18), comparative analysis of virulence factors (19), and genome analysis and sequence retrieval (20).

BioCyc is a collection of Pathway/Genome databases also known as PGDBs (21). Each PGDB is created specifically for a single organism/strain. The web-based version, which is publicly available at biocyc.org, contains a series of tools for navigating, visualizing and analyzing the PGDBs and for omics data analyses. Among the major features implemented within each PGDB are (i) a genome browser, (ii) gene ontology, pathway ontology, enzyme commission ontology, and compound ontology browsers, (iii) a tool for visualization of metabolic pathways and metabolic maps, (iv) tools for identification of chokepoint reactions and dead-end metabolites, (v) a regulatory overview tool for visualization of the regulatory interactions, (vi) a tool for user-generated omics data analysis by overlapping of the data onto metabolic maps, regulatory interaction maps and genome maps, (vii) the possibility to create an account to store groups of genes and pathways, called SmartTables, with sharing capabilities and quick access, (viii) several comparative analysis tools across PGDBs, (ix) a comprehensive search tool that allows for searching across peptide and nucleotide patterns as well as annotated features, and (x) BLAST functionality directly from the PGDB web page. The PGDBs can be classified into three groups depending on the level of manual curation. Tier 1 databases are intensively curated, Tier 2 databases are computationally derived databases with moderate curation, and Tier 3 databases are computationally derived databases subjected to no curation.

Here, we describe the first *L. monocytogenes* computer-assisted and manually curated (Tier 2) database implemented within the framework of the Pathway Tools software and hosted within the BioCyc web site (22). The database aims to provide scientists with a resource that allows access to various features such as protein function, gene ontologies, reactions, metabolic pathways, regulatory interactions, operon information, regulatory entities, and bibliographical references. Although not all the data has been manually curated, there is an ongoing effort to have the entire database manually curated and updated at least yearly. Users of the database can also upload their own omics data to generate figures and graphs in the context of the regulatory interactions, reactions and pathways in which their genes of interest are involved. Moreover, gene lists can be uploaded and used for gene enrichment analyses within the website.

### Implementation

The *Listeria monocytogenes* strain 10403S PGDB (referred as the *L. monocytogenes* 10403S PGDB hereon) was constructed based on the 10403S finished genome sequence. The genome was sequenced in a collaborative effort including Cornell University, UC Berkeley, Harvard Medical School, the Broad Institute, the Centers for Disease Control and Prevention (CDC), Cerus Corporation, and Institut Pasteur and released on March 24, 2010 as part of the *Listeria monocytogenes* sequencing project [*Listeria monocytogenes* Sequencing Project, Broad Institute of Harvard and MIT (20)]. Two independent annotations were used as input to generate the PGDB; an annotation released with the genome sequence as part of the *L. monocytogenes* sequencing project (20) and an annotation generated using the Rapid Annotation using Subsystem Technology (RAST) Server (23). The CDS products from these two annotations were combined into a single GenBank format file and further analyzed. For CDSs where one of the annotations had a hypothetical protein designation for the product variable but the other annotation had a more meaningful designation for this same variable, the hypothetical protein designation was discarded. For CDSs where both designations were similar, one of them was discarded. In general, the CDS products annotated by RAST were used with Pathway Tools. The initial database was generated by the PathoLogic component of the program Pathway Tools version 14.0 and has been updated upon new releases of Pathway Tools. Briefly, PathoLogic was used to convert the *L. monocytogenes* 10403S genome file in a GenBank format into a PGDB by transforming gene and gene product descriptions into a PGDB representation. Then, PathoLogic was used to predict the metabolic pathways carried out by the *L. monocytogenes* strain 10403S from its genome by comparison with the MetaCyc PGDB (24). After the automated creation of the *L. monocytogenes* 10403S PGDB, a manual refinement was carried out using PathoLogic, which allows for (i) definition of certain enzymes that could not be unambiguously called during the automated process, (ii) rescoring and rebuilding of pathways based on the refined annotation of new enzymes, (iii) manual creation of protein complexes suggested by the annotation, (iv) operon assignment and (v) other fine-tuning operation. Then, a consistency check was performed to verify for possible broken links across objects within the database, formatting errors, and validity of citations. Manual curation of the database was carried out using a series of tools for identification and annotation of genes and protein functions, literature mining and a combination of published (25) and unpublished RNA sequencing (RNA-Seq) data. Briefly, for each curated gene, a bibliographical search was carried out to identify previous studies that had characterized the gene in *L. monocytogenes*, other *Listeria* species, other Firmicutes or any other prokaryotic organism. If no publication could be found, *in silico* analyses were carried out using NCBI BLAST (26), the UniProtKB (27) and the EMBL-EBI InterProScan web tool (28) for identification of protein functions, the AmiGO toolkit (29) for annotation of GO terms, the ENZYME database (30) for identification and annotation of reactions and pathways, and the MetaCyc, EcoCyc and BsubCyc databases for general annotation. Transcription start sites were manually identified based on directional RNA-Seq data or based on published data and annotated accordingly within the PGDB. Promoter regions were manually identified upstream of transcription start sites identified through directional RNA-Seq data based on consensus sequence similarity or based on published data and annotated accordingly. Regulatory interactions were annotated based on published data or based on σ factors promoters and transcription factor sequences identified upstream the transcription units.

The *L. monocytogenes* 10403S PGDB has information available for all 2979 genes including 2828 protein-coding genes, and 151 RNA genes. Among those 151 RNA genes, 18 encode for ribosomal RNAs (rRNAs), 85 encode for transporter RNAs (tRNAs) and 48 encode other noncoding RNAs (ncRNAs). A total of 1729 transcription units were annotated in a genome size of 2 903 106 bases. A total of 207 pathways can be accessed along with 1173 enzymatic reactions and 119 transport reactions. One pathway, the d-chiro-inositol degradation pathway, along with five reactions and four compounds within the pathway were created specifically for this database. Out of the 2828 protein-encoding genes, 961 encode for enzymes (not including genes with unknown function, which may also encode for enzymes) and 233 encode transporters. Three protein annotations were specifically created for this database. A total of 62 proteins contain comments and 71 contain citations with direct links to PubMed. Gene ontology terms (GO terms) are provided for 877 proteins. A total of 262 proteins and genes have been curated as on 09 July 2014. Currently, the database also provides the curated localization and sequence information for 67 σ^A^-dependent promoters, 75 σ^B^-dependent promoters, 2 σ^L^-dependent promoters and 1 σ^H^-dependent promoter for a total of 145 promoter regions. Moreover, the database provides information on 34 curated DNA binding sites for 12 different transcription factors, including all described-to-date DNA binding sites for the positive regulator of *L. monocytogenes* virulence genes, PrfA, the motility regulators MogR and DegU and the stress response regulators CtsR and HrcA. There are a total of 788 citations in the *L. monocytogenes* 10403S PGDB, which include citations for genes, proteins and pathways.

### Usage

The *L. monocytogenes* 10403S PGDB can be used for genome browsing and to obtain information on regulators and regulatees, gene ontology, transcription units, reactions and pathways. Users will find a wide range of tools including a regulatory interaction map, which shows the regulatory network involving σ factors and transcription factors currently curated, and a cellular overview, which shows all reactions and pathways potentially taking place in the cell based on genome annotation and curation. In comparison to the *L. monocytogenes* strain EGD-e BioCyc database (*L. monocytogenes* EGD-e PGDB), which has gone through no curation, the *L. monocytogenes* 10403S PGDB has 77 more pathways, including 148 biosynthetic pathways in the *L. monocytogenes* 10403S PGDB versus 93 biosynthetic pathways in the *L. monocytogenes* EGD-e PGDB, 78 pathways involved in degradation, utilization or assimilation in the 10403S database versus 48 of such pathways in the *L. monocytogenes* EGD-e PGDB and 25 pathways involved in generation of precursor metabolites and energy in the 10403S database versus 18 of such pathways in the *L. monocytogenes* EGD-e PGDB. Moreover, the *L. monocytogenes* 10403S PGDB has 233 transporters listed compared to 130, in the EGD-e database. Users will benefit from using the *L. monocytogenes* 10403S PGDB instead of the *L. monocytogenes* EGD-e PGDB when accessing the regulatory content of the *L. monocytogenes* 10403S PGDB. Because regulatory information is only added to the databases by curation (i.e. no computational automated process creates regulation-related information), the *L. monocytogenes* EGD-e PGDB has no information concerning regulation of genes, RNA or proteins. Conversely, the *L. monocytogenes* 10403S PGDB includes 191 regulatory interactions, 146 promoters, 14 proteins annotated as transcription factors and 176 transcription factor binding sites.

**Figure 1.**
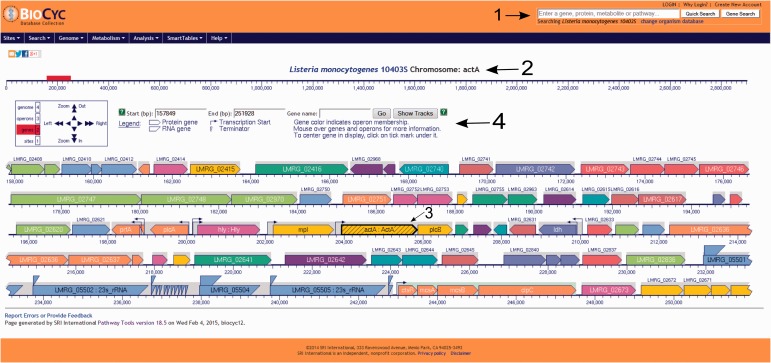
Screenshot of the *L. monocytogenes* 10403S PGDB genome browser tool. Users can run a quick search for gene names, gene locus, protein name, pathways, reactions or compounds, can login to their private account or create one, which allows the creation and utilization of groups to be analyzed within the database and change the database to be viewed (arrow 1). In the example shown here, the virulence gene *actA* was selected (arrow 2) and is centered and highlighted within the genome browser window (arrow 3). On top of the genome browser window, users can zoom in and out, move upstream (left) or downstream (right) in the chromosome, select a specific coordinate or gene and have a legend explaining the differences across colors and shapes (arrow 4). Contiguous genes with the same color are part of the same transcription unit (i.e. operon). Transcription start sites are shown as arrows upstream the respective transcription units.

**Figure 2.**
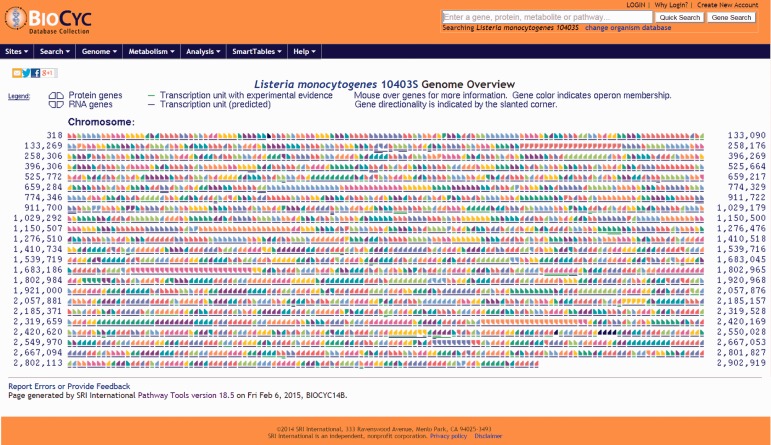
Screenshot of the genome overview page. All annotated genes and genetic features are shown. Consecutive genes sharing the same color are part of the same transcription unit (i.e. operon) and share a contiguous underline. Chromosome coordinates are shown on the left and right sides of the page. All items are clickable and take the user to the corresponding genetic feature (e.g. gene, tRNA, rRNA) page.

**Figure 3.**
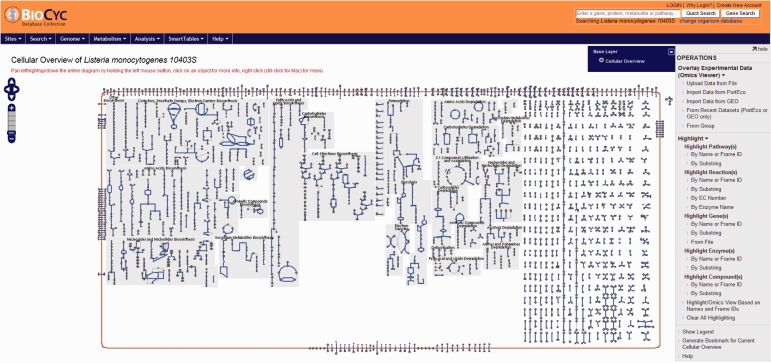
Screenshot of the cellular overview page. The graphic represents all reactions, including transport of compounds across the membrane, predicted to be carried out by the cell. Reactions are represented by blue lines. Different compounds are represented by different geometrical shapes. The compounds are: amino acids (pointing up triangles); carbohydrates (squares); proteins (diamonds); purines (vertical ellipses); pyrimidines (horizontal ellipses); cofactors (pointing down triangles); tRNAs (tee); and other (circles). Transport reactions are shown along the cell membrane (as reddish rectangle). Pathways are shown with background gray shading. Reactions that do not belong to a specific pathway are shown at the right side of the picture with no background gray shading.

**Figure 4.**
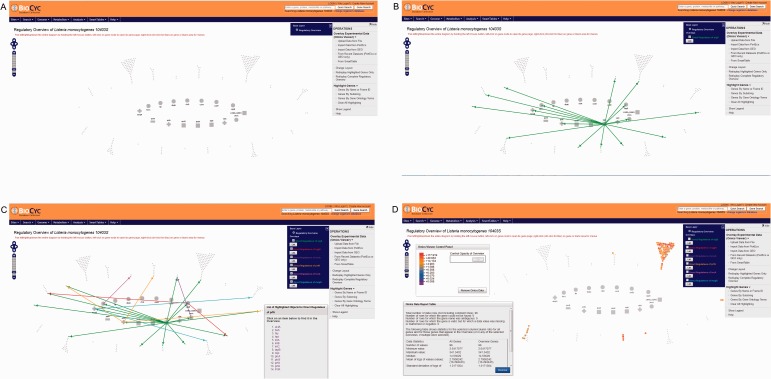
Screenshots of the regulatory overview page showing different options. The inner ring contains master regulators and σ factors while the outer ring contains genes that are regulated only. Genes that are solely regulated by activators are represented by a plus sign. Genes that are solely regulated by inhibitors are represented by a minus sign. Genes that regulated by both inhibitors and activators are represented by a circle. Genes encoding σ factors are represented by squares. LMRG_02663 encodes the alternative σ factor, σ^H^. (**A**) Initial page showing the “Operations” menu at the right. (**B**) Screenshot showing genes directly regulated by σ^B^ highlighted in green. Notice that among these genes are the transcription factor encoding genes *prfA*, *mogR*, *yvoA*, *hrcA* and *cggR*. C: Screenshot showing genes directly and indirectly regulated by σ^B^. Genes are highlighted with different colors according to the transcription factor that directly regulates them; genes directly regulated by σ^B^ are highlighted in green. Genes indirectly regulated by σ^B^ and directly regulated by other regulators are color coded in orange (PrfA-regulated genes), light blue (MogR-regulated genes), purple (YvoA-regulated genes), red (CggR-regulated genes) and magenta (HrcA-regulated genes). A list of genes directly regulated by PrfA is shown at the bottom right. (**D**) Screenshot showing the overlay of experimental data within the regulatory overview. The average fold changes (FC = parent strain/Δ*sigB* mutant) of significantly σ^B^ positively regulated genes identified in an RNA-Seq study by (25) were overlaid. A legend at the top left part of the figure shows the color-coding associated with the amplitude of the FC. A “Omics Data Report Table” providing some summary statistics is also shown at the bottom left part of the screenshot. Notice that most genes identified in that study as being σ^B^-dependent are solely regulated by σ^B^.

Users have the option to run a quick search for gene name, locus name, protein name, ontologies, pathway, reaction or compound or run an advanced search where several options are available such as DNA binding sites, regulation and citations. Every search result will provide a link that will take the user to the corresponding webpage. Users can also use the genome browser tool (Figure 1) to navigate through the chromosome and access any gene or genetic feature displayed. Similarly, users can access all genes in the chromosome through the genome overview tool (Figure 2). Pathways, reactions and compounds can also be easily accessed through the cellular overview (Figure 3), which displays a map of all pathways and reactions predicted to be carried out by the strain based on genome content and manually curated published data. From the cellular overview, users also have access to links to membrane proteins such as ABC transporters and internalin proteins. Through the regulatory overview (Figure 4), users can access regulatory networks involving manually curated proteins in the genome. For example, the user can select to display the regulators of a giving protein or the regulatees (i.e. protein regulated) of a giving transcription factor or σ factor. All these tools provide quick and easy access to specific information on the proteins and genes that might be of interest to the user.

**Figure 5.**
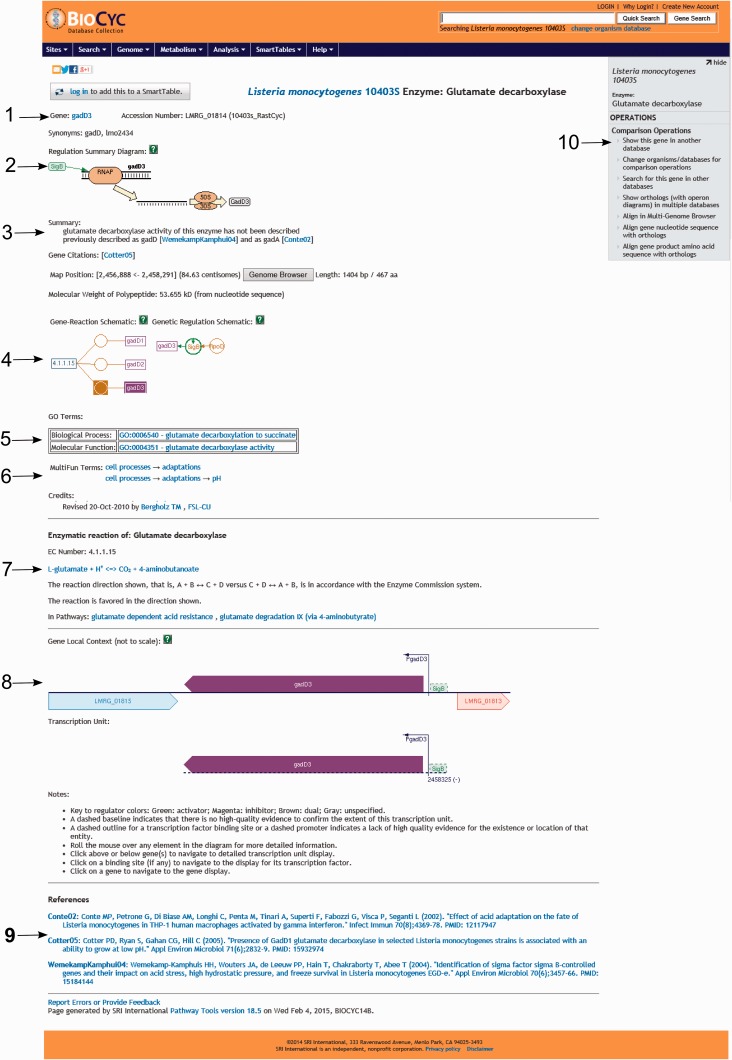
Screenshot of the gene *gadD3* page. The page includes several useful pieces of information such as (1) enzyme name, gene name, gene locus name and synonymous names; (2) a summary diagram of the expression regulation; (3) a summary text with pertinent information associated with the gene or protein, literature citations, genome localization, and protein molecular weight; (4) schematics representing the reactions carried out by the enzyme and the regulatory interactions involved in the gene expression regulation; (5) Gene ontology terms associated with the protein function; (6) MultiFun terms associated with the protein function and credits; (7) information on the reaction(s) carried out by the enzyme, including the pathways (if any) where this reaction may occur; (8) a diagram representing the region where the gene is located in the genome, including neighboring genes, promoters and regulatory regions and the transcription unit(s) associated with the gene; (9) bibliographical references with links to NCBI PubMed and (10) an “Operations” box with several options for comparative genomics analyses using the gene.

For genes that were manually annotated, which can be identified by the credit at the bottom of the page, users will find several useful pieces of information including (i) gene name and gene accession number (i.e. locus name in strain 10403S); (ii) protein name; (iii) alternative protein name for selected proteins; (iv) gene locus name of the ortholog in strain EGD-e; (v) a regulation summary diagram showing the transcription regulators of the gene itself as well as other genes in the same operon; (vi) citations with direct links to NCBI PubMed, (vii) protein localization, map position, gene/protein length; (viii) predicted (from sequence) and experimental (when available) molecular weight of the protein; (ix) a genetic regulation schematic showing the regulatory interactions involving the regulators and regulatees of the selected gene; (x) GO terms associated with the selected protein; (xi) MultiFun terms, a Pathway Tools built-in ontology schema; (xii) a graphical representation of the genomic region surrounding the selected gene, including neighbor genes, transcription start sites and regulators involved in transcription regulation of the selected gene; (xiii) graphical representation of transcription units in which the selected gene is transcribed (i.e. if a gene is transcribed monocistronically and bicistronically, both transcription units are shown); (xiv) a legend for understanding of the graphical representations and full references with links to NCBI PubMed (Figure 5).

## New insights into *L. monocytogenes* physiology

The *L. monocytogenes* BioCyc database annotation revealed that the *L. monocytogenes* genome codifies for all enzymes necessary for the utilization of ethanolamine and propanediol. Ethanolamine, a source of carbon and nitrogen, is present in eukaryotic cells (31) and may be available during host infection or food contamination. Propanediol is produced during the catabolism of rhamnose and fucose, two sugar that can be found in some foods, such as smoked salmon (32). *L. monocytogenes* also harbors all the enzymes necessary for biosynthesis of adenosylcobalamin (vitamin B_12_), a cofactor necessary for both ethanolamine and propanediol degradation (33). The ability to synthesize its own vitamin B_12_ and to utilize ethanolamine and propanediol may give *L. monocytogenes* a selective advantage against other microorganisms during food contamination and host infection.

### Conclusions

Here, we present the first partially curated (Tier 2) *L. monocytogenes* BioCyc database, which has been created based on annotation of the strain 10403S genome. This database has many advantages over other noncurated *L. monocytogenes* databases, including a larger number of annotated pathways and reactions, and transporters as well as additional information on regulation, which is completely absent from noncurated databases. Moreover, the database is hosted within BioCyc, a widely used collection of databases with multiple functionalities allowing users to access and interact with the data and also analyze their own “omics” data using the resources available in the database. The *L. monocytogenes* 10403S PGDB is under continuous curation and updates to the database will be released yearly. We expect to curate all the genes in the 10403S genome by 2015, while keeping the already curated genes updated as more information becomes available through publications. New information that will be added to the database will include data on transcription terminators, noncoding RNAs, post-translational regulation, and availability of knock-out mutants for selected genes.

## Availability and Requirements

The web version of the *L. monocytogenes* strain 10403S BioCyc database can be accessed from the BioCyc website (22) by selecting the *Listeria monocytogenes* 10403S database under ‘change organism database’. The 10403S BioCyc database is also available for download with the Pathway Tools program from (34). Pathway Tools is available for Linux/x86, Windows/x86 and Macintosh.
